# Chaperones contribute to G protein coupled receptor oligomerization, but do not participate in assembly of the G protein with the receptor signaling complex

**DOI:** 10.1186/1750-2187-5-16

**Published:** 2010-09-24

**Authors:** Maha M Hammad, Denis J Dupré

**Affiliations:** 1 Department of Pharmacology, Faculty of Medicine, Dalhousie University, Halifax, NS, Canada

## Abstract

**Background:**

Previous studies have demonstrated that seven transmembrane receptors (7TM-Rs) can associate with various chaperones to control their maturation and export. It has been shown for a few years now that 7TM-Rs can form homo or heterooligomeric complexes. Due to the difficulty to study heterooligomers in a context devoid of homooligomers signaling, very little is known on heterooligomerization. β2AR-AT1R receptor complexes have been found on cells and ligand activation of one receptor affects signaling of the partner. Yet, very little is known about the mechanisms linking those receptors together. We propose to examine the role of chaperones in the maturation of homo- and heterodimers of the β2AR and AT1R. It would not be surprising that strict cellular mechanisms exist to ensure that only properly folded receptors are inserted into the plasma membrane.

**Results:**

Our goal is to understand the process whereby the adrenergic and angiotensin receptors attain their proper mature conformation. We determined whether any of the common chaperones are physically associated with the fully and/or immature β2AR and AT1R receptors forms and if they play any role in the selective recruitment of G proteins subunits to receptor complexes. Our results suggest that when a pair of receptors is expressed in such way that one is retained in the endoplasmic reticulum (ER), this immature receptor will dictate the chaperones interacting with the receptor complex. We showed that ERp57 is important for receptor dimerization of AT1R homo and β2AR/AT1R receptor dimers, but plays no role in the β2AR homodimerization. Then, we verified if some of those chaperones could play a role in the assembly of the heterotrimeric G protein subunits with the receptor complex, but none appeared to be essential.

**Conclusions:**

Overall, our results suggest that variations among receptor oligomers occur early in the synthesis/maturation processes, and that chaperones will interact more specifically with some receptor pairs than others to allow the formation of certain receptor pairs, while others will contribute to the folding and maturation of receptors without any effect on receptor assembly within a signaling complex.

## Background

Seven transmembrane receptors (7TM-Rs) are the largest family of plasma membrane receptors and couple to G proteins to activate downstream signaling pathways that give rise to alterations in cell function and gene expression [1]. These receptors had traditionally been thought to exist as monomers, but it is now known that 7TM-Rs exist as homo- and heterooligomers [2,3]. Heteromerization of receptors is an exciting new field in pharmacology. It is possible that drugs that act on one receptor in a heteromer might influence the signal transduction mechanisms activated by the other receptor. AT1R undergoes homo- and heterodimerize with many other receptors, including bradykinin B2, β2-adrenergic and dopamine D2 receptors [4-7]. Until recently, the functional consequence of heterodimerization had not been described and its functional significance was questioned. Now, examples of heterodimerization show that this phenomenon is clinically relevant and should be taken into account during therapy. For example, heterodimerization of prostaglandin EP_1 _receptors with β_2_ARs [8] showed that where EP_1 _receptors do not appear to significantly affect airway tone, it was able reduce the bronchodilatory function of β_2_-ARs. The functional significance of 7TM-R heterodimers has also recently been demonstrated *in vivo *for opioid receptors, for which a heterodimer-selective agonist was described, consistent with the hypothesis that these complexes occur in native tissues [9]. Interestingly, it is known that AT1Rs heterodimerize with many other receptors, including β2ARs, where it was also demonstrated that blockade of one of the two receptors in complex was enough to inhibit signaling and trafficking of both receptors. Treatment of murine myocytes with a β blocker completely obliterated Angiotensin receptor/Gq coupling and contractility, and treatment of mice with a selective angiotensin receptor blocker attenuated heart rate response to a β agonist [7].

Effects like this could contribute to the adverse effects reported in Congestive Heart Failure patients taking a combinatorial therapy including AT1R blockers and βAR blockers [10]. However, little is known about the formation of receptor heterodimers and the factors that are important in the formation of AT1R/β2AR heterodimers have not been established. Precise characterization of receptor-mediated signaling pathways will be crucial for the development of new therapeutic targets outside the receptor-ligand interface. Drugs aimed at the ligand binding site of 7TM-Rs that are coupled to multiple effectors obviously lack specificity and activate many different effector routes. This can activate multiple signaling pathways and generate unwanted side effects. However, the presence of unique signaling partners within a particular pathway (for example, G proteins with a specific subunit composition, chaperones or other regulatory molecules, GTPases, scaffolds or effectors) suggests that there are unique structural components that govern interactions between these proteins.

Previous studies have demonstrated that non-glycosylated immature AT1R can associate with chaperones such as calnexin and HSP70, while ER mannosidase I participates in the acquisition of mature glycoforms of the AT1R [11]. AT1R can also interact with DRiP78, a DnaJ family protein known to retain proteins by masking ER-export sites before they are fully folded and ready to be sent to the plasma membrane [12]. In contrast, very little is known about the chaperones which interact with the β2AR. Recently, it was demonstrated that DRiP78, a DnaJ class chaperone, could interact with the β2AR and the Gγ subunit of the G protein [13]. It is possible that DRiP78 and other chaperones are involved in the formation of β2AR/AT1R receptor complexes. We propose to examine the role of chaperones in the maturation of homo- and heterodimers of the β2AR and AT1R. Due to the complex structure and organization of these receptors, it is not altogether surprising that strict cellular mechanisms exist to ensure that only properly folded receptors are inserted into the plasma membrane. 7TM-Rs are known to undergo multiple steps of folding and posttranslational modifications including N- and O-glycosylation, disulfide bonds and palmitoylation which are regulated by yet undetermined chaperones. Yet, it is not clear which chaperones might be involved in the processing of 7-TMRs such as the β2AR and AT1R. Our goal is to understand the process whereby the adrenergic and angiotensin receptors attain their proper mature conformation and to determine how these receptors are assembled into homo and heterodimeric receptors. In these experiments, we will determine whether any of the commonly used protein-folding chaperones are physically associated with the fully and/or immature β2AR and AT1R receptors forms and if they play any role in the selective recruitment of G proteins subunits to receptor complexes.

## Results and Discussion

7TM-Rs are known to undergo multiple steps of folding and post-translational modifications including N- and O-glycosylation, disulfide bonds and palmitoylation which allows proper expression and functions of receptors. These steps are highly regulated and occur before receptors are exported to plasma membrane. The overall processes regulating 7TM-R exit from the ER and trafficking to plasma membrane are not very well known. In recent years, some studies have started to address the key issues on how receptors traffic to plasma membrane, and how they are assembled into signaling complexes [14]. Some chaperones have been shown to play an important role in the folding and assembly of different members of the GPCR signaling complex [13,15,16]. While previous studies have demonstrated that non-glycosylated immature AT1R can associate with various chaperones, it is not clear which ones might be involved in the assembly of 7-TMRs into signaling complexes; if any could be important in the formation of receptor oligomers, or assembly of the receptor with its cognate G protein and therefore contribute to signaling specificity of receptor complexes.

### BiFC Visualization of 7-TMRs homo and heterodimers

**Figure 1:**
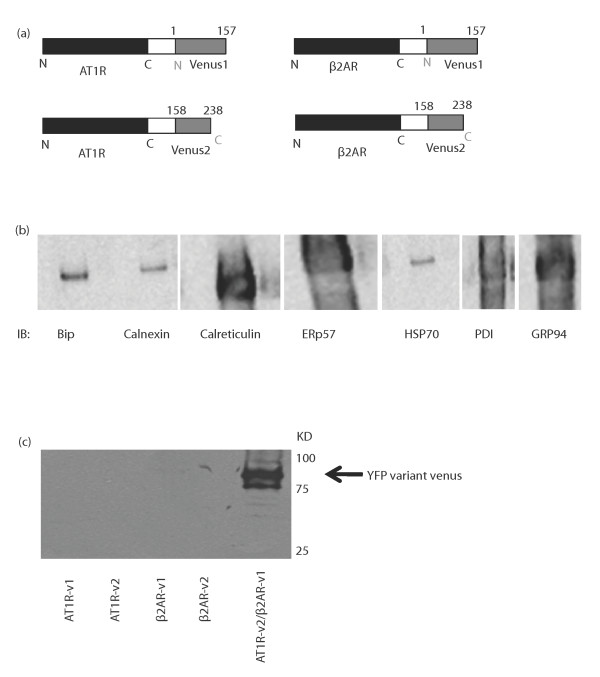
**Expression of the receptor dimer pairs**. a) Schematic presentation of the constructs used in the experiments. Venus1 contains the N- terminal end of the yellow fluorescent protein variant (venus) and represents the first 157 amino acids while venus2 contains the C- terminal end of the protein and represents amino acids 158 to 238. b) HEK293 cells were harvested, washed and lysed with RIPA buffer. Samples were then run on SDS-PAGE and Western analysis was performed using indicated chaperone antibodies (1:2000). c) Specificity of GFP antibody to dimers was tested in HEK293 cells. The cells were transfected with AT1R-v1 or v2, β2AR-v1 or v2 and AT1R-v2 with β2AR-v1. 48 hours after transfection, cells were lysed in RIPA buffer. Western analysis was performed using anti-GFP (1:2000) and indicate the presence of a yellow fluorescent protein variant, venus. Results are representative of 3 independent experiments.

The study of heterodimeric receptors is still in its infancy, given the difficulty of studying those receptors without the background of homodimers expressed in the same cells. Up to now, most studies revealing interactions between receptors was done via co-immunoprecipitation or via fluorescence based protein-protein interaction techniques such as Fluorescence Resonance Energy Transfer (FRET) or Bioluminescence Resonance Energy Transfer (BRET). Here, we used another fluorescence based technique, Bimolecular Fluorescence complementation (BiFC), as a tool to obtain a fluorescence signal when two receptors dimerize. The advantage of using this technique is that it allows another fluorescent- or bioluminescent-tagged protein to be used in FRET or BRET, therefore allowing to monitor the specific interaction of a given combination of receptor pair with other signaling partners. To do so, we tagged our β2-adrenergic and angiotensin II AT1 receptors with the first 157 amino acids of venus, a YFP variant, or with the 158-238 remaining amino acids of venus (Figure 1a). As previously described for BiFC [17], expression of each construction individually does not produce fluorescence, while expression of both halves, when paired to interacting proteins, will generate a functional fluorescent protein.

In order to visualize the expression of the different receptor pairs, we performed imaging where HEK293 cells were transfected with the indicated receptor pairs in Additional file 1: figure S1 (a-k). 48 hours after transfection, the slides were fixed with 3% paraformaldehyde for 20 mins and then fixed on cover slips. Upon receptor dimerization, the venus molecule reconstitutes and emit fluorescence at 525 nm when excited at 488 nm. The first three figures (a-c) show the expression of wild type receptor pairs that are either in a homodimer arrangement (AT1R and β2AR or in the heterodimer form AT1R/β2AR). As illustrated, the receptors are expressed at the plasma membrane and they are lining the surface of the cell. In contrast, when at least one of the receptors in the dimer is immature i.e. has a mutation at the glycosylation sites, the receptors are retained inside the cell. Figure 1(d-j) shows that the strong fluorescence signal is mainly concentrated in the endoplasmic reticulum region which indicates that the trafficking of mutant receptors to the plasma membrane is abolished. In addition, these immature receptors prevent the wild type forms from being expressed at the plasma membrane as well. This is consistent with the results obtained with the dopamine D2 receptor [18], CCR5 chemokine receptor [19] and Vasopressin V2 receptor [20] where co expressing a mutant receptor with the wild type form resulted in the blockade of cell surface expression of the dimer. Figure (1k and 1l) show intracellular localization of receptor pairs expressing a receptor mutated in the FX_(6)_LL motif. Our results confirm the intracellular retention of such mutated receptors, and that such mutation prevents export of WT receptors to plasma membrane as well.

### Chaperone interaction with 7TM-Rs homo and heterodimers

Interactions with ER-resident folding factors begin typically, as growing nascent chains enter the ER lumen through the translocon complex and continue until the newly synthesized protein is fully folded and assembled. It is known that chaperones function to assist other proteins to achieve proper folding [21-26]. The rules that direct the selection of the different chaperones required for proper folding and assembly of newly synthesized proteins are only very poorly understood. Different groups of chaperones have been identified, each with a particular mode of action: 1) A group of classical chaperones that include BiP and several glucose-related proteins (GRPs) represent a bona fide chaperone class. They stabilize newly synthesized polypeptides during folding, prevent aggregation and mediate retention of peptides in the ER and suppress formation of non-native disulfide bonds [27]. 2) A second group of chaperones, such as the lectins, have greater substrate specificity. They are non-enzymatic sugar-binding proteins and are represented in the ER by calnexin and calreticulin. It has been suggested that all glycoproteins (such as 7TM receptors) interact with ER lectins in mammalian cells [28]. 3) Other proteins with chaperone activity directly increase the rate of peptide folding, such as the peptidyl-prolyl isomerase family that facilitates the rotation around peptidyl-prolyl bonds of nascent poplypeptides [29,30]. The folding catalysts also include enzymes of the protein-disulfide isomerase (PDI) family, which are responsible for the formation of disulfide bonds [31-33]. The interplay between these different chaperone classes will determine the processes involved in folding each protein.

**Figure 2:**
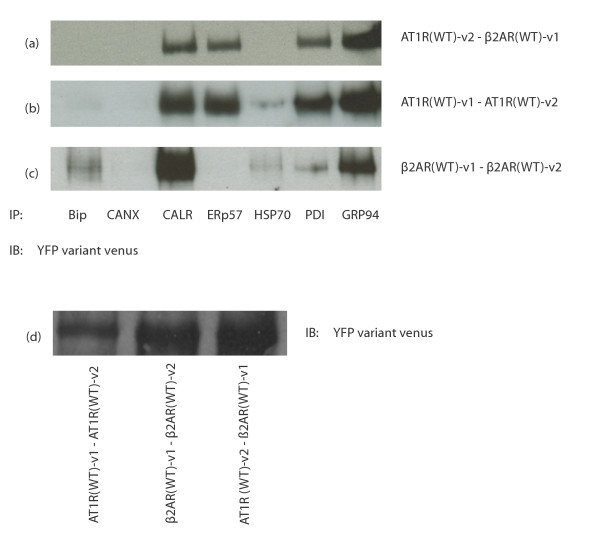
**Interaction of wild type receptor dimers with chaperones**. HEK293 cells were transfected with AT1R (WT)-v1/v2 and β2AR (WT)-v1/v2. After 48 hours, cells were harvested, washed, lysed with RIPA. This lysate was distributed into eight different microcentrifuge tubes and co-immunoprecipitations were performed using the indicated chaperone antibody. a) AT1R/β2AR (WT) heterodimer, b) AT1R (WT) homodimer c) β2AR (WT) homodimer. The eighth sample was loaded as a control to show the expression level of the wild type receptor dimers as shown in d. Results are representative of 4 independent experiments.

Very little is known about the chaperones which interact with the β2AR. Here, we propose to determine which chaperones are involved in the maturation of homo- and heterodimers of the β2AR and AT1R. First, we co-expressed the WT isoforms of β2AR and AT1R, each tagged with a portion of venus, as previously demonstrated in Figure 1. Hek293 cells were tested for the expression of each chaperone tested (figure 1b) and Bip, Calnexin, Calreticulin, ERp57, HSP70, PDI and GRP94 were all expressed. Then, we proceeded with immunoprecipitation of those chaperones and immunoblot against GFP, which would reveal only the functional GFP, observed only upon dimerization of the GPCR pairs. Receptors expressed with only a portion of the venus would not be recognized by the anti-GFP antibody we used (figure 1c). Figure 2 shows the interaction patterns of previously mentioned chaperones with the β2AR-venus1/AT1R-venus2 dimer (figure 2a), AT1R-venus1/AT1R-venus2 dimer (figure 2b) and β2AR-venus1/β2AR-venus2 dimer (figure 2c). Although interactions with calreticulin, PDI and GRP94 appear to be conserved through all receptor pairs, differences in the interaction pattern can be observed in the case of Bip, which interacts only with the β2AR homodimer, ERp57 which interacts only with AT1R-containing dimers, and HSP70 which interacts weakly with both homodimers, but not the heterodimer. A reason for the differences observed for HSP70 interaction with both homodimers and not the heterodimer might be related to some observations made about heterooligomeric receptors. For example, a heterodimeric receptor could couple to G- protein subunits other than those recruited by the homodimers and hence activates effectors differently. This was suggested for the mu/delta opioid heterodimer where no sensitivity to pertussis toxin was observed while the homodimers of these receptors were sensitive to it [34]. This was also illustrated for the chemokine receptors CCR5/CCR2 as well where the heterodimer was shown to couple to Gq/11, a subunit that does not interact with CCR5 homodimer or CCR2 homodimer [35]. Our results might suggest that very early on, during receptor maturation, begins the differentiation of the receptor complex formation which will lead to the variations in receptor signaling between homo and heterooligomeric receptors. When doing the co-immunoprecipitation, all culture plates transfected with the same cDNAs were pooled together and then co-immunoprecipitations were done with each anti-chaperone antibody. Figure 2d shows the expression of receptors, demonstrating that we can achieve good expression of the receptor complexes in HEK293 cells.

**Figure 3:**
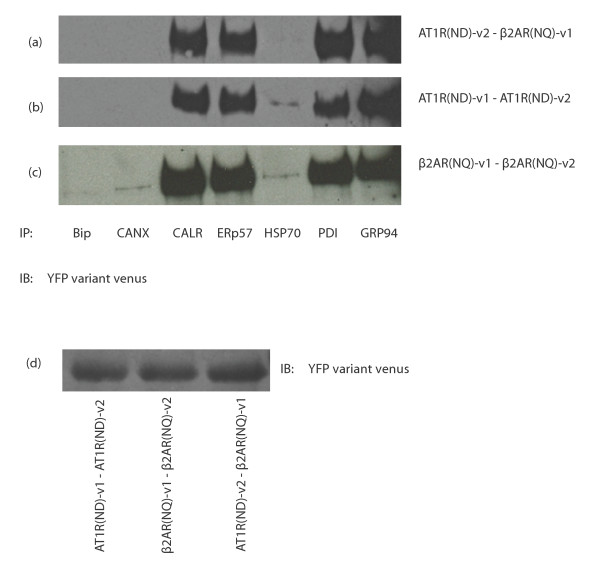
**Interaction of immature receptor dimers with chaperones**. HEK293 cells were transfected with AT1R (N4, 176, 188D)-v1/v2 and β2AR (N 4, 15, 176Q)-v1/v2. After 48 hours, cells were harvested, washed, lysed with RIPA and precleared with protein A-sepharose beads. This lysate was distributed into eight different microcentrifuge tubes and co-immunoprecipitations were performed using the indicated chaperone antibody. a) AT1R (ND)/β2AR (NQ) heterodimer, b) AT1R (ND) homodimer, c) β2AR (NQ) homodimer. The eighth sample was loaded as a control to show the expression level of the immature receptor dimers as shown in d. Results are representative of 4 independent experiments.

As show in additional file 1: figure S1, the co-expressed WT receptor pairs follow the entire process of maturation to be ultimately expressed at the plasma membrane. We wanted to understand whether the interaction of chaperones required fully mature receptors, or if the chaperones would interact with receptors that lack some glycosylation sites, which would prevent the receptors from reaching plasma membrane. Here, we used AT1R with mutations at sites N4, 176, 188 D and β2AR with mutations at positions N 4, 15, 176Q to understand the effect of those glycosylation sites on chaperone interactions with the receptor dimers. Interestingly, some variations happen in the chaperone interaction pattern with receptor dimers following expression of the glycosylation mutated receptors (Figure 3). Striking examples include the appearance of interaction of ERp57, calnexin with the β2AR homodimer (figure 3c), which was absent upon co-expression of WT receptors (figure 2c). Also, the weak interaction of PDI with the β2AR dimer gained strength with the non-glycosylated mutants. Those results suggest that these chaperones are important for the maturation into the glycosylated forms of the receptor. Interestingly, Bip interaction was lost with the non-glycosylated form of the β2AR. No change was observed for any chaperone with the AT1R receptor homodimer, or the β2AR/AT1R dimer. It appears that for those pairs, the chaperone interaction profile follows the one needed for AT1R, which might act as the dominant receptor of the pair in terms of maturation.

**Figure 4:**
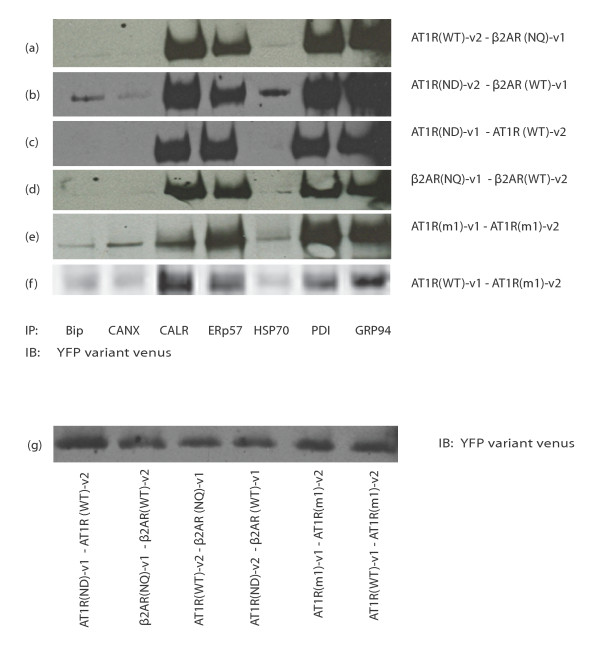
**Immature receptors dictate dimers interaction with chaperones**. HEK293 cells were transfected with AT1R (WT or ND)-v1/v2 and β2AR (WT or NQ)-v1/v2. After 48 hours, cells were harvested, washed, lysed with RIPA and precleared with protein A-sepharose beads. The lysate was distributed into eight different microcentrifuge tubes and co-immunoprecipitations were performed using the indicated chaperone antibody. The eighth sample was loaded as a control to show the expression level of the different receptor dimers as shown in g. Results are representative of 4 independent experiments.

We then moved to co-express receptor pairs where one receptor would be able to fully mature (WT), with a partner which would be glycosylation deficient, to see the influence of receptors on each other's maturation and chaperone interaction. We show in figure 4 (figure 4a, b, c, and 4d) that the chaperone interaction pattern follows exactly the pattern observed previously for the least mature receptor. Those results suggest that the least mature receptor would retain the WT receptor inside the cell as previously shown in the additional figure. This would be logical since the effects of having an immature receptor expressed at plasma membrane could have important consequences on the signal transduction. Figure 4 rows e and f show the influence of a mutant of AT1R which is retained in the ER (AT1Rm), but has no effect on glycosylation. The mutation is in the F(X)_6_LL motif, where the F and LL are mutated to alanine. This motif has been shown to be important for export from the ER for several receptors [36,37]. Again, the least mature receptor dictates the interaction pattern with chaperones, as observed in rows 4e and 4f, where the ER-retained receptor AT1Rm/AT1R WT pair has the same interaction pattern as the AT1Rm/AT1Rm pair. Interestingly, with the AT1Rm, all chaperones tested show some level of interaction with the receptor complex. Since this is a receptor mutation that would not be able to be changed by chaperone intervention, there is most likely a complete blockade of the maturation pathway which would result in receptor dimer interaction with chaperones at each step of the pathway. This could explain the interaction with all chaperones, assuming that their actions do not occur all at once on the receptor, but rather more or less consecutively as literature suggests. Figure 4g shows that all receptor pairs could be recognized by our anti-GFP antibody.

### Effect of Chaperones on WT receptor dimerization

**Figure 5:**
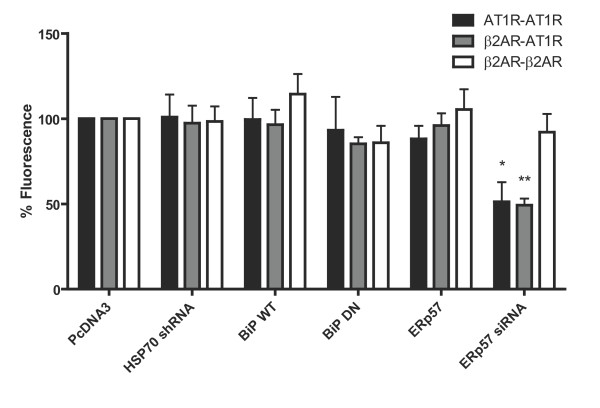
**The effect of Bip, HSP70 and ERp57 on receptor dimerization**. HEK293 cells were transfected with WT AT1R-v1/v2 and β2AR-v1/v2. After 48 hours, cells were harvested, washed with 1X PBS and resuspended in 100 μl PBS. Fluorescence was then measured using an EnVision plate reader at 528 nm. Results are expressed as means ± sem of at least 3 experiments. * = *p *< 0.05; ** = *p *< 0.01 compared with negative controls using two-tailed paired Student's *t *test. Results are representative of 3 independent experiments.

It is already known that several chaperones are required for the assembly of different protein complexes. For example, Phosducin-like protein (PhLP) and dopamine receptor interacting protein 78 (DRiP78) were shown to form a complex in order to assemble the Gβγ subunits of the G protein [15,16,38]. In other cases, chaperones were able to interact with channels, but played no role in the proper assembly and folding of the channel. One example would be calnexin, which interacted transiently with wild-type Shaker protein in the ER but, failed to associate with an unglycosylated Shaker mutant that makes active, cell surface channels. Glycosylation of Shaker protein was required for association with calnexin, but was not required for the proper folding and assembly of Shaker channels [39]. Here, we tested the effect of chaperones on receptor dimerization. In order to obtain a functional venus, which is able to produce fluorescence, the receptor dimers needs to be assembled. We co-expressed different chaperone WT or mutants constructs (Bip WT or T37G), or inhibitory RNA to observe the effects of chaperones on fluorescence formation. We tested the effect of WT Bip, calnexin, GRP94 and ERp57 and none showed a significant modification of the level of fluorescence observed (Figure 5 for Bip and ERp57, data not shown for calnexin and GRP94). We were intrigued to know if overexpressing a chaperone would promote the formation of dimers, which was not the case for the chaperones tested. This suggests that there probably already are sufficient levels of chaperones in the cell to deal with any increased demand for protein production. Then, we tested the effect of lowering the levels of functional chaperones expressed with inhibitory RNA (Efficiency of inhibition shown in additional file 2: Figure S2) or a mutated inactive form of Bip. No change were observed with the Bip mutant either. ERp57 shRNA co-expression with the β2AR homodimer showed no difference, but significant changes in fluorescence levels were observed upon co-expression with the AT1R homodimer or with β2AR/AT1R heterodimer. Such role for ERp57 wouldn't be exclusive for 7TM-Rs, as it was shown the MHC class I receptors that a calnexin/ERp57 complex is thought to facilitate the correct oxidation and folding of class I heavy chains into a conformation compatible with recognition by β2m [40].

### Effect of Chaperones on G protein assembly with receptors

**Figure 6:**
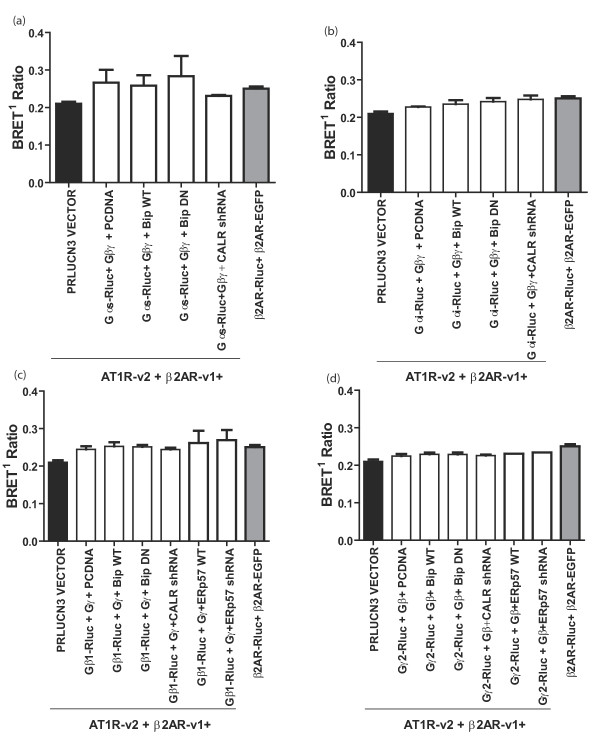
**The effect of Bip, Calreticulin and ERp57 on G-protein assembly with the heterodimer**. HEK293 cells were transfectedwith β2AR-v1, AT1R-v2 and Rluc G-proteins (Gαs, Gαi, Gβ1 and Gγ2). 48 hours post transfection, cells were harvested, washed three times with PBS then suspended in 100 μl PBS. 90 μl of the samples was distributed into 96 well plates and 10 μl of 5 μM coelenterazine H was added and mixed then the fluorescence energy emitted by Rluc and YFP (venus) was measured at their corresponding emission wavelengths (460 and 528 nm, respectively). The BRET ratio was then calculated and plotted with the ratios for a negative control (β2AR-v1, AT1R-v2 and the pRluc-N3 vector) and a positive control (β2AR-Rluc and β2AR-EGFP). Results are expressed as means ± sem of at least 3 experiments. Statistical analysis was performed using two-tailed paired Student's *t *test.

It was shown that some chaperones have an effect on the assembly of G proteins coupling to 7TM-Rs. Several studies have shown that a complex of chaperones, formed by PhLP, the cytosolic chaperonin complex (CCT) would interact with the beta subunit of the G protein, while DRiP78 would interact with the gamma subunits of the G protein prior they are assembled together [15,16,38]. Results showed, at least for DRiP78, that it stabilized Gγ before the stable interaction with Gβ [38]. It was also demonstrated that the G protein subunit could form a complex with the receptor before it reaches plasma membrane [14]. Here, we tested whether some of the chaperones capable of interacting with the β2AR and AT1R receptors could mediate the formation of the receptor-G protein complex. We tested the effect of Bip, calreticulin and ERp57 on the assembly of Gα subunits, as well as the Gβγ subunit with the receptors AT1R and β2AR homodimers, and AT1R/β2AR heterodimer as measured by Bioluminescence Resonance Energy Transfer (BRET). Figure 6 shows results from the AT1R/β2AR heterodimer (AT1R and β2AR homodimers results are not shown) which suggests that none of the chaperones tested actually have an effect on the assembly of the G protein with the receptor dimers. We co-expressed along with the venus-tagged receptor dimers the Galphas-Rluc or Galphai-Rluc subunits, in presence of Gbeta1gamma2, therefore forming a functional G protein with the receptor pair. Then, cells were harvested and treated with coelentrazine H and measurements of BRET signals were taken. Signals of luciferase luminescence and fluorescence from venus were then used to make a BRET ratio. The BRET ratio gives an indication of the interaction between the different partners. No effect was observed for the different chaperones tested (figure 6a and 6b). The same experiment was repeated in absence of Galpha subunits, as it was previously demonstrated that Gβγ could interact with the β2AR under such circumstances. Again, none of the chaperones tested had a significant impact on the BRET signal observed between the venus-tagged receptor dimer and the Rluc-tagged G protein subunits Rluc-Gβ1 or Rluc-Gγ2 (in presence of the untagged other subunit to form a stable Gβγ complex) (figure 6c and 6d). Our results demonstrate that none of the chaperones tested have an impact on the formation of the G protein association with the receptor. Although these chaperones have no impact, it doesn't mean that there wouldn't be a contribution of chaperones in the assembly of the receptor signaling complex. Proteins that may be considered chaperones for G protein subunits like Gα include the J domain-containing cysteine string protein (CSP) [41], Gα-interacting vesicle-associated protein (GIV, also known as girdin), Daple, and FLJ000354 [42]. These proteins may be involved in assembling G protein heterotrimers and may also represent potential interacting proteins for PhLP-1 and/or DRiP78. The fact that certain chaperones such as CSP and DRiP78 can also interact with effector molecules [e.g., voltage-gated calcium channels [43-45] or GPCRs (e.g., dopamine D1, M2 muscarinic cholinergic, angiotensin II AT_1 _receptors [12,46,47], and the β_2_AR [38]) suggests that these chaperones may also be involved in the formation or trafficking of specific receptor signaling complexes that include the G protein heterotrimer. Overall, more work is needed to fully understand the receptor signaling complex assembly with its cognate G protein, and how specificity of signal transduction can be attained.

## Conclusions

Molecular chaperones are highly important for the maturation and folding of signaling proteins such as plasma membrane receptors and effectors. In this report, we investigated the role of several chaperones that are commonly found to interact with a variety of partners. We show that not all chaperones will interact in a similar fashion with 7TM-R dimers, whether they are homo or heterodimeric. Our results suggest that when a pair of receptors is expressed in such way that one is retained in the ER or not fully glycosylated, this immature receptor will dictate the chaperones interacting with the receptor complex. We showed that ERp57 is important for receptor dimerization of AT1R homo and β2AR/AT1R receptor dimers, but plays no role in the β2AR homodimerization. Then, we verified if some of those chaperones could play a role in the assembly of the heterotrimeric G protein subunits with the receptor complex, but none appeared to be essential. Overall, our results suggest that variations among receptor oligomers occurs early in the synthesis/maturation processes, as chaperones will interact more specifically with some receptor pairs than others. We propose that other chaperones, potentially chaperones playing a more specific role, might act upon the receptor and G proteins to create functional signaling complexes. Yet, no chaperone has been demonstrated to play such role but their identification could provide new therapeutic targets to control the levels of receptor signaling complex expression in cells.

## Methods

### Reagents

Reagents were obtained from the following sources: fetal bovine serum and Lipofectamine 2000 transfection reagent were from Invitrogen (Etobicoke, ON, Canada); Dulbecco's modified Eagle's medium high glucose and all chemicals were obtained from Sigma-Aldrich (Oakville, ON, Canada), unless noted. Bip, ERP57, calnexin, calreticulin, HSP70, PDI and GRP94 antibodies were purchased from Cedarlane Labs (ON, Canada) and polyclonal GFP antibodies were purchased from Santa Cruz (Santa Cruz, CA, USA). Calreticulin and ERp57 inhibitory RNAs were obtained from Applied BioSystems (Carlsbad, California, USA). HSP70 inhibitory RNA was purchased from Santa Cruz (Santa Cruz, CA, USA).

### Constructs

All BRET constructs (controls, as well as G protein subunits) were obtained from Dr. Terence E. Hébert (McGill University). β2AR and AT1R F(X)_6_LL mutants were obtained from Guangyu Wu (Lousiana State University Health Sciences Center) while AT1R ND-GFP mutant was obtained from Dr. Gaétan Guillemette (Université de Sherbrooke). AT1R N4,176,188 D venus1 and AT1R N4,176,188 D venus2 were cloned by amplifying AT1RND-GFP by PCR using AT1R ND FWD primer (5'-AAGCTGCTAGCATTCTCGACTCTTCTACTGAAGATGGT-3') and AT1R RVS primer (5'-GCCACCTTCGAACTCAACCTCAAAACATGGTGCAGGCTT-3'). The PCR product was subcloned into either pcDNA3.1 zip-venus1 or pcDNA3.1 venus2 vectors using NheI-ClaI/BstBI as the restriction sites. β2AR N4, 15, 176Q amplified by PCR using β2AR (NQ) FWD primer (5'-ATGTGCGGCCGCACCATGGGGCAACCCGGGCAGGGC-3') and β2AR (NQ) RVS primer (5'-GCCACCATCGATCAGCAGTGAGTCATTTGT-3') and subcloned into pcDNA3.1 vectors containing either zip-venus1 or venus2 using NotI-ClaI sites.

### Cell Culture and Transfections

HEK293 cells were grown in Dulbecco's modified Eagle's medium high glucose supplemented with 10% fetal bovine serum and transfected using Lipofectamine 2000 as per the manufacturer's instructions. Cells were plated in 6-well plates. Experiments were carried out 48 hours after transfection.

### Immunoprecipitation

48 hours after transfection into 100-mm^2 ^dishes (for these experiments 4 μg of each cDNA was transfected into each dish, and total DNA levels/dish were kept constant by adding pcDNA vector as required). Cells were then washed with PBS and harvested. Cells expressing the same cDNAs were pooled together and then evenly distributed in microcentrifuge tubes for co-immunoprecipitations. Samples were lysed in 0.8 ml of radioimmune precipitation assay (RIPA) buffer (50 mM Tris, pH 7.5, 10 mM MgCl_2_, 150 mM NaCl, 0.5% sodium deoxycholate, 1% Nonidet P-40, 0.1% SDS, Complete Protease inhibitors (Roche; Laval, QC, Canada) and DNase I). The lysate was solubilized by incubation at 4°C for 30 min, precleared with 50 μl of protein A-Sepharose beads at 4°C for 1 h, and clarified by centrifugation at 14,000 rpm for 10 min. Supernatants were then transferred into another microcentrifuge tube and incubated with an antibody overnight. The immunoprecipitated proteins were eluted from beads with 50 μl of SDS sample buffer and resolved by SDS-PAGE, and Western blots were performed as described [48]. When immunoprecipitation was not required, cells were lysed in 200 μl of RIPA buffer, precleared with protein A-sepharose and then SDS-PAGE loading buffer was added. Immunoblots were probed with either a monoclonal anti-GFP antibody (1:1000) or monoclonal anti-HA (Covance, 1:1000 dilution), Horseradish peroxidase-conjugated secondary antibodies were also from Santa Cruz (anti-mouse or anti-rabbit, 1:10,000).

### Bimolecular Fluorescence Complementation (BiFC) and Bioluminescence Resonance Energy Transfer (BRET)

HEK293 cells were co-transfected with vectors expressing the GFP- and *R*luc-fusion proteins (1 μg of each cDNA was transfected into each well of a 6-well plate, and total DNA/dish was kept constant by adding pcDNA vector as required). 48 hours after transfection, cells were harvested and washed once with phosphate-buffered saline (PBS). The cells were then suspended in PBS+ (PBS + 0.1% glucose) and distributed into 96-well microplates (white Optiplate; Perkin-Elmer Life and Analytical Sciences). Signals were collected on a Packard Fusion instrument (Perkin- Elmer Life and Analytical Sciences) using coelenterazine H as a substrate. Whether or not BRET occurred was determined by calculating the ratio of the light passed by the 450/58 (luciferase) and 535/25-nm band pass filters (YFP) for BRET1. This ratio is referred to as the BRET ratio. To avoid possible variations in the BRET signal resulting from fluctuation in the relative expression levels of the energy donor and acceptor, we designed transfection conditions to maintain constant GFP/*R*luc expression ratios in each experimental set. BiFC signals were determined by the measurement of the light that passed by the 535/25-nm band pass filters (YFP). BRET background was determined under conditions where resonance energy transfer between *R*luc and GFP either could not or did not occur. This was accomplished by expressing *R*luc or *R*luc-tagged proteins either alone or together with GFP or GFP-tagged proteins, none of which interact physiologically. The background was the same regardless of which of the aforementioned individual proteins or combinations of proteins were expressed, and it has been subtracted to yield a BRET Ratio.

### Fluorescence Microscopy

Twenty four hours post-transfection, HEK 293 cells were harvested and seeded on laminin-coated cover slips for 4 h at 37°C. The cells were then fixed for 20 min in PBS, pH 7.4, containing 3% (w/v) paraformaldehyde. The cover slips were washed with PBS, drained, and mounted onto glass slides using a drop of 0.4% 1,4-diazabicyclo{2.2.2}octane/glycerol medium. Cover slips were fixed to the slides with nail polish. Fluorescence microscopy was performed with an Olympus IX81 equipped with a Photometrics coolSNAP HQ2 camera and excite series 120Q light source. YFP (venus) was excited at 488 nm, and image acquisition was done at fluorescence emission 525 nm.

## Competing interests

The authors declare that they have no competing interests.

## Authors' contributions

MMH carried out most of the experiments described in the study and contributed to draft the manuscript. DJD conceived the study, and participated in its design and coordination and helped to draft the manuscript. All authors read and approved the final manuscript.

## Supplementary Material

Additional file 1 **figure S1**. Images a through l were obtained by transfecting HEK293 cells with the indicated receptors and mounting the cover slips on glass slides. Visualization was performed using an inverted fluorescence microscope (Olympus IX81), a) AT1R-v1/AT1R-v2, b) β2AR-v1/β2AR-v2, c) AT1R-v2/β2AR-v1, d)AT1R (ND)-v1/AT1R (ND)-v2, e) β2AR (NQ)-v1/β2AR (NQ)-v2, f)AT1R (ND)-v2/β2AR (NQ)-v1, g) AT1R-v2/β2AR (NQ)-v1, h)AT1R (ND)-v2/β2AR-v1, i)AT1R-v1/AT1R (ND)-v2, j) β2AR-v1/β2AR (NQ)-v2, k)AT1Rm1-v1/AT1Rm1-v2, l)AT1R-v1/AT1Rm1-v2. Click here for file

Additional file 2 **figure S2**. HEK293 cells transfected or not with HSP70 shRNA, calreticulin or ERp57 siRNAs were harvested 24 hours post-transfection. Cells were then lysed in RIPA buffer and samples were loaded on SDS-PAGE gel for analysis of the presence of each chaperone in the lysate using the appropriate antibody recognizing the chaperone. Results are representative of at least 3 experiments, each performed individually. Click here for file
