# Supplementary figures and images for: Chaperones contribute to G protein coupled receptor oligomerization, but do not participate in assembly of the G protein with the receptor signaling complex
Source: J Mol Signal. 2010 Sep 24;5:16. doi: 10.1186/1750-2187-5-16 (PMC2954983; doi:10.1186/1750-2187-5-16)

Supplemental Figure 1

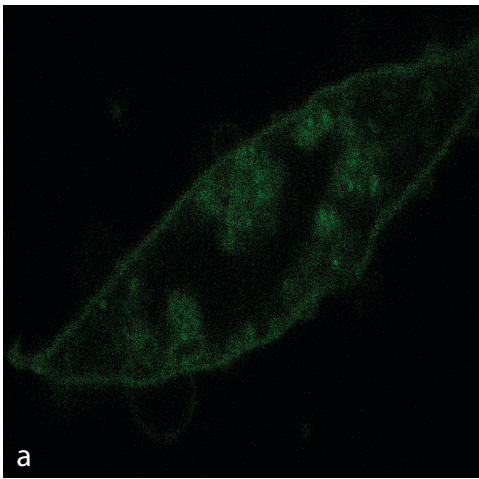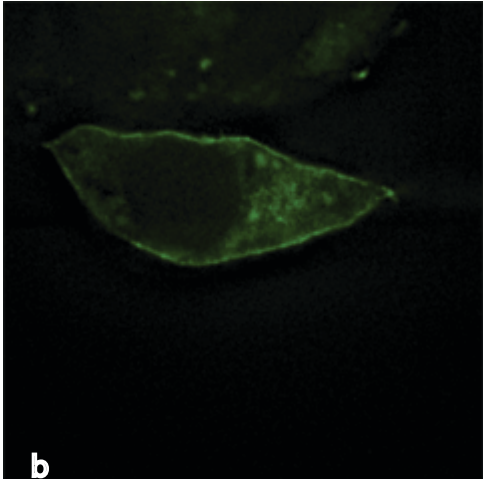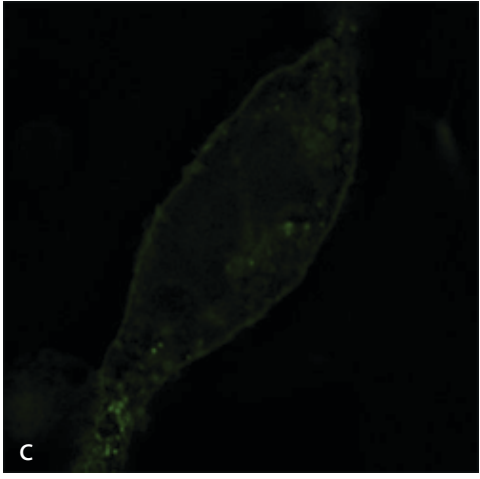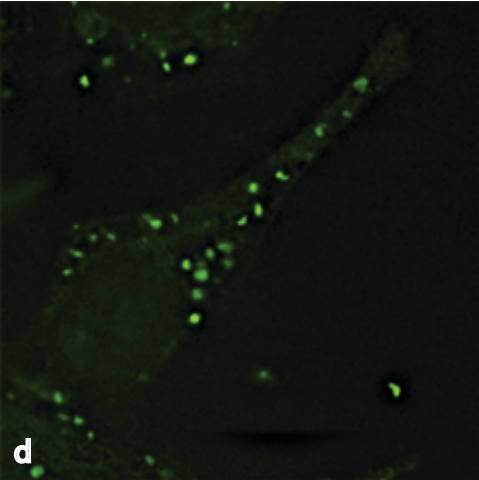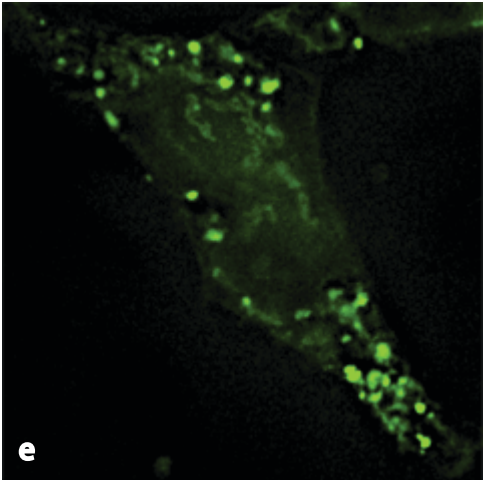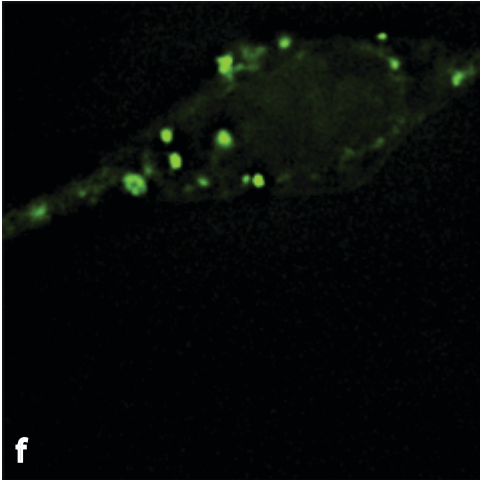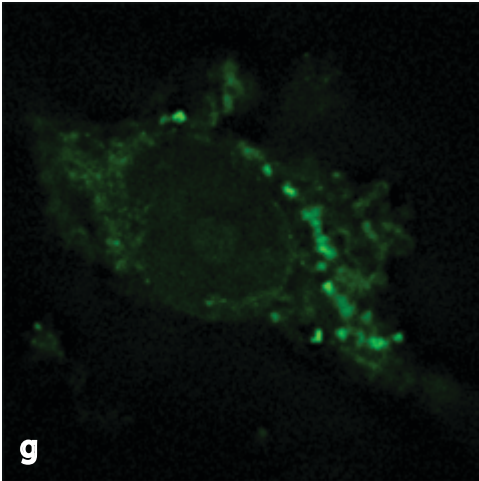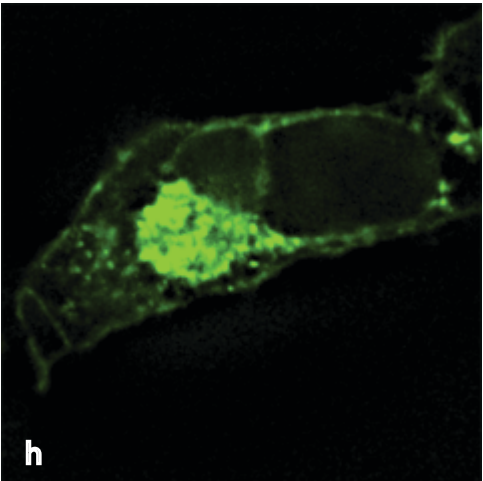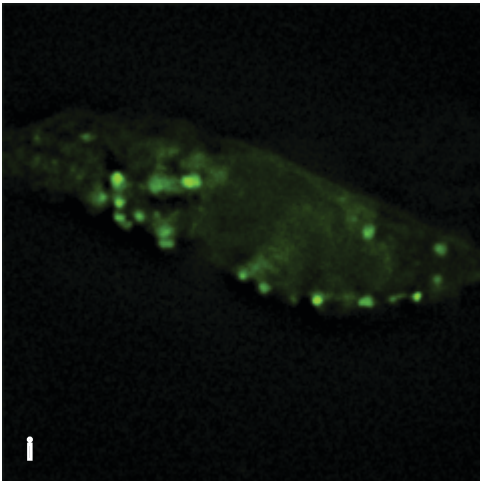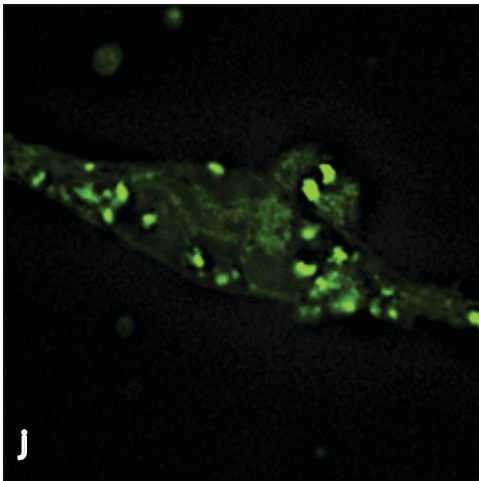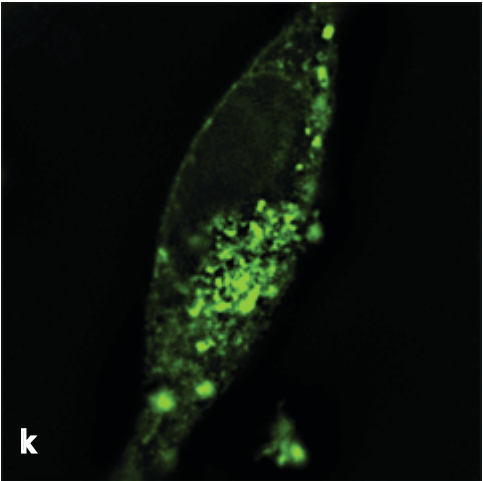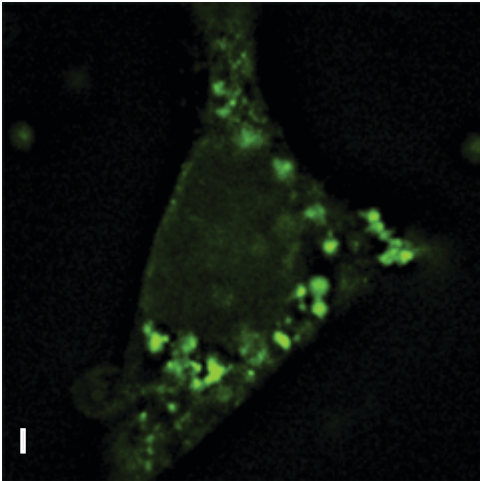

Supplement: Additional file 1 — figure S1. Images a through l were obtained by transfecting HEK293 cells with the indicated receptors and mounting the cover slips on glass slides. Visualization was performed using an inverted fluorescence microscope (Olympus IX81), a) AT1R-v1/AT1R-v2, b) β2AR-v1/β2AR-v2, c) AT1R-v2/β2AR-v1, d)AT1R (ND)-v1/AT1R (ND)-v2, e) β2AR (NQ)-v1/β2AR (NQ)-v2, f)AT1R (ND)-v2/β2AR (NQ)-v1, g) AT1R-v2/β2AR (NQ)-v1, h)AT1R (ND)-v2/β2AR-v1, i)AT1R-v1/AT1R (ND)-v2, j) β2AR-v1/β2AR (NQ)-v2, k)AT1Rm1-v1/AT1Rm1-v2, l)AT1R-v1/AT1Rm1-v2. [file 1750-2187-5-16-S1.PDF]

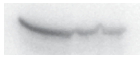

- +  
HSP70  
shRNA

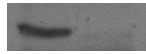

- +  
ERp57  
siRNA

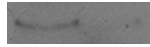

- +  
calreticulin  
siRNA

HEK293 cell lysate

Supplement: Additional file 2 — figure S2. HEK293 cells transfected or not with HSP70 shRNA, calreticulin or ERp57 siRNAs were harvested 24 hours post-transfection. Cells were then lysed in RIPA buffer and samples were loaded on SDS-PAGE gel for analysis of the presence of each chaperone in the lysate using the appropriate antibody recognizing the chaperone. Results are representative of at least 3 experiments, each performed individually. [file 1750-2187-5-16-S2.PDF]
